# 3,4,5,4′-*trans*-tetramethoxystilbene (DMU-212) modulates the activation of NF-κB, AP-1, and STAT3 transcription factors in rat liver carcinogenesis induced by initiation-promotion regimen

**DOI:** 10.1007/s11010-014-1983-9

**Published:** 2014-02-13

**Authors:** Michał Cichocki, Wanda Baer-Dubowska, Marcin Wierzchowski, Marek Murias, Jadwiga Jodynis-Liebert

**Affiliations:** 1 Department of Pharmaceutical Biochemistry, Poznan University of Medical Sciences, Swiecickiego 4 Str., 60-781 Poznań, Poland; 2 Department of Chemical Technology of Drugs, Poznan University of Medical Sciences, Grunwaldzka 6 Str., 60-780 Poznań, Poland; 3 Department of Toxicology, Poznan University of Medical Sciences, Dojazd 30 Str., 60-631 Poznań, Poland

**Keywords:** 3,4,5,4′-tetramethoxystilbene (DMU-212), N-nitrosodiethylamine, Liver carcinogenesis, NF-κB, AP-1, STAT3

## Abstract

It has been reported that methylated analog of resveratrol, 3,4,5,4′-*trans*-tetramethoxystilbene (DMU-212), demonstrates strong antiproliferative, and proapoptotic activity. The aim of this study was to evaluate the effect of DMU-212 on the activation of nuclear factor-κB (NF-κB), activator protein-1 (AP-1), and signal transducer and activator of transcription 3 (STAT3) transcription factors, using a two-stage model of rat hepatocarcinogenesis (HCC) in Wistar rats. Initiation was performed by a single intraperitoneal injection of N-nitrosodiethylamine (NDEA) (200 mg/kg) followed by promotion with phenobarbital (PB) (0.05 %) in drinking water. DMU-212 was administered by gavage in a dose of 20 or 50 mg/kg b.w. two times a week for 16 weeks. There was a significant increase in the activation of all investigated hepatic transcription factors in the NDEA/PB-induced rats. The activation of NF-κB induced by NDEA/PB treatment was suppressed by DMU-212 as evidenced by a reduction of p65 and p50 subunits translocation, DNA binding capacity, increased retention of IκB, and the reduced IKK activity. Moreover, DMU-212 reduced the level of iNOS protein induced by NDEA/PB. Treatment with DMU-212 alone increased the constitutive AP-1 subunits c-Jun and c-Fos levels and c-Jun binding to TRE consensus site. The combined treatment diminished c-Fos level and DNA binding. At a dose of 50 mg/kg, DMU-212 decreased also the STAT3 activation induced by NDEA/PB. These data indicate that DMU-212 may suppress pro-inflammatory transcription factors, particularly NF-κB, and in consequence iNOS expression in rat model of HCC which makes DMU-212 a good candidate for the development of HCC chemopreventive agent.

**Electronic supplementary material:**

The online version of this article (doi:10.1007/s11010-014-1983-9) contains supplementary material, which is available to authorized users.

## Introduction

Resveratrol (3, 4′, 5-trihydroxystilbene), a naturally occurring phytoalexin found in grapes, red wines, berries and peanuts, has been reported to show a variety of beneficial properties including antioxidative and anticarcinogenic ones [1, 2]. Resveratrol has been demonstrated to affect a multitude of signal transduction pathways associated with tumorigenesis [3], thus the pleotropic activity, rather than just a single effect, seems to play an important role in the potential anticancer properties of stilbenoids. These promising data have encouraged the search for resveratrol’s derivatives showing enhanced biological activity as well as improved pharmacokinetic parameters. Compared to resveratrol, its analogs with ortho-methoxy substituents have been found to be more potent in some in vitro and in vivo studies. Thus such derivatives, including *trans*-3,4,5,4′-tetramethoxystilbene (DMU-212), may serve as a useful starting point for the design of improved chemopreventive or cancer therapeutic agents [4]. Our earlier studies have shown that this derivative exerts stronger antiproliferative and proapoptotic activity than the parent compound in ovarian cancer cells culture in vitro [5]. Similar observations have been also reported by other researchers in different cell systems [4, 6].

Proapototic activity is often linked to enhanced activation of nuclear factor kappa B (NF-κB) and activator protein-1 (AP-1). These transcription factors as well as signal transducers and activators of transcription 3 (STAT3) are constitutively active in many kinds of cancers including liver cancer, and play critical roles in tumor development and progression through modulation of their target genes involved in angiogenesis, metastasis, and cell survival [7, 8]. Moreover, STAT3 is considered as the marker of metastasis [9].

Liver cancer, predominantly hepatocellular carcinoma (HCC), is a complex and fatal malignancy, driven primarily by oxidative stress and inflammation. Because of the lack of effective diagnostic tools for early detection and limited treatment options available to patients with advanced stages of HCC, there is a critical need for discovery and development of novel preventive and therapeutic strategies to combat the current morbidity and mortality associated with HCC [10].

Resveratrol has been shown to prevent N-nitrosodiethylamine (NDEA) initiated hepatocarcinogenesis in rats through suppression of inflammation and oxidative stress [11, 12]. Resveratrol-mediated chemoprevention of rat liver carcinogenesis has been suggested to be related to alteration of proinflammatory cytokines [12]. A number of transcription factors have been connected to the inflammation and oxidative stress. Among them, the most important are NF-κB and AP-1. Activation of NF-κB leads to transcription of numerous genes including those encoding for expression of inflammatory cytokines, and cyclooxygenase-2 (COX-2) [13]. Another proinflammatory protein/enzyme transcriptionally regulated by NF-κB and/or AP-1 is inducible nitric oxide synthase, iNOS [14].

In view of the above, the aim of our current study was to investigate whether DMU-212, a methylated resveratrol analog, may exert chemopreventive, anti-apoptotic, and antinflammatory effects through modulation of transcription factors NF-κB, AP-1, and STAT3 induced by NDEA/PB regimen in rat liver.

## Materials and methods

### Materials

3,4,5,4′-*trans*-tetramethoxystilbene (DMU-212) was synthesized by partial methylation of *trans*-resveratrol as described elsewhere. Its structure and purity was confirmed by means of gas chromatography–mass spectrometry and nuclear magnetic resonance spectroscopy [15].

Rabbit polyclonal p50, p65, c-Jun, c-Fos, iNOS, COX-2, JNK, IκBα, IKKα/β, and pSTAT3 antibodies, anti-rabbit IgG-alkaline phosphatase (AP) conjugated antibody, and anti-rabbit IgG-horseradish peroxidase (HRP) conjugated antibody were supplied by Santa Cruz Biotechnology (Santa Cruz, CA, USA). SDS-PAGE Gels (5–10 %) and Western blotting detection system were purchased from Bio-Rad Laboratories (Hercules, CA, USA). AP-1 and NF-κB activation tests were supplied from Active Motif (Carlsbad, CA, USA). Substrate peptides for IKK activity assay were purchased from GL Biochem (Shanghai, China). NDEA and phenobarbital (PB) were provided by Sigma-Aldrich Poland, 2-hydroxypropyl-β-cyclodextrin by Bonio Inc., Canada.

### Animals and treatment

Two stage model of hepatocarcinogenesis, initiation by NDEA and promotion by phenobarbital, was applied. Male Wistar rats, 240 ± 10 % *g*, bred at the Department of Toxicology, Poznan University of Medical Sciences were used. The rats were housed in animal facility, at 22 ± 2 °C with a 12 h light/dark cycle, controlled humidity and circulation of air, and fed certified laboratory feed (Labofeed H, ISO 22000). Animals were divided randomly into 5 groups, 8 animals each and subjected to the following treatment. Rats in group II were administered DMU-212 by gavage at a dose 50 mg/kg b.w. two days a week for 16 weeks. To improve bioavailability, the compound was suspended in the 40 % solution of 2-hydroxypropyl-β-cyclodextrin [16]. Groups III, IV, and V were given intraperitoneally a single dose of NDEA, 200 mg/kg b.w., followed by promotion with phenobarbital at a concentration of 0.05 % in drinking water. Promotion was started 2 weeks after NDEA injection. Rats in groups IV and V were administered DMU-212 by gavage at doses 20 and 50 mg/kg b.w., respectively, two days a week for 16 consecutive weeks. Rats in group I (controls) were given vehicle by gavage (40 % solution of 2-hydroxypropyl-β-cyclodextrin) two days a week for 16 weeks.

After 16 weeks following NDEA injection, the animals were anesthetized by ketamine and xylazine, the blood was withdrawn from the heart, livers were excised, rinsed with ice-cold 1.5 % KCl, and stored in −70 °C until isolation of extracts or subcellular fractions.

The experiment was performed according to the Local Animal Ethics Committee guidelines for animal experimentation.

### Preparation of liver extracts

The pre-cut livers were placed in Total Protein Isolation Buffer (TPIB; 50 mM Tris–HCl, pH 7.4, 150 mM NaCl, 1 % NP-40, 0.1 % SDS, 1 mM EDTA, 1 mM PMSF, 1 mM Na_3_VO_4_, 1 mM NaF), containing protease inhibitors (leupeptin and aprotinin, 1 μg/ml each), homogenized in ice-chilled glass homogenizer with Teflon pestle, lysed for 40 min on ice with vortex mixing every 10 min, and centrifuged at 15,000 *g* for 30 min at 4 °C. Supernatants were collected, assayed for protein concentration using the Lowry method, aliqoted and stored at −70 °C until used for analysis.

### Preparation of nuclear and cytosolic extracts

Nuclear and cytosolic extracts were prepared using Nuclear/Cytosol Fractionation Kit (BioVision Research) according to the manufacturer’s instructions. Briefly, liver homogenate was centrifuged at 500 g for 5 min at 4 °C. Pellets were resuspended in an ice-cold cytosol extraction buffer containing dithiothreitol (DTT) and protease inhibitors. After incubation in an ice bath for 10 min, 11 μl of Cytosol Extraction Buffer B were added, the samples were mixed and then centrifuged at 16,000 *g* for 5 min 4 °C to collect the cytosolic fractions. The supernatants (cytosolic fractions) were transferred into clean tubes. The pellets were resuspended in an ice-cold nuclear extraction buffer containing DTT and protease inhibitors and incubated on ice for 40 min with vortex mixing for 15 s every 10 min. The lysed suspension of nuclei was then centrifuged at 16,000 *g* at 4 °C for 10 min, and the supernatants were collected as nuclear fractions. The collected cytosolic and nuclear fractions were assayed for protein concentration using the Lowry method, aliquoted and stored at −70 °C until used for Western blot or ELISA analysis.

### Western blot analysis

For the analysis of protein level, the total cell extracts or subcellular fractions were boiled in loading buffer (2.7 M Tris–HCl, 20 % SDS, 80 % glycerol, 250 mM DTT, 0.01 % bromophenol blue). Thirty micrograms of the sample protein were resolved on polyacrylamide gels (Biorad). The resolved proteins were transferred to a PVDF membrane (Millipore). The blot containing the transferred protein was blocked in a blocking buffer (10 % fat-free milk in DPBS-T, containing 10 mM Tris–HCl, pH 7.6, 150 mM NaCl, and 0.1 % Tween-20). The blots were then incubated for 2 h with primary antibodies dissolved in DPBS-T, washed three times and subsequently incubated for 1 h with secondary antibodies conjugated with alkaline phosphatase. After washing three times with DPBS-T and two times with TBS (20 mM Tris–HCl, 500 mM NaCl; pH 7.4), the blots were placed in 0.1 M Tris buffer (pH 9.5), and proteins were detected by means of Alkaline Phosphatase Conjugate Substrate Kit (BioRad Laboratories). Beta-actin was used as an internal control. The amount of immunoreactive product in each line was determined by densitometric scanning using a Biorad Quantity One software. The values were calculated as relative absorbance units (RQ) per mg of protein.

### NF-κB, AP-1, and STAT3: DNA binding assays

NF-κB, AP-1, and STAT3 activation was assessed by an enzymatic immunoassay according to Renard et al. [17] using the commercial kits (TransAM assays; Active Motif, Carlsbad CA, USA) and following the manufacturer’s instructions. Activated NF-κB was measured in terms of the amount of p65, and AP-1 in terms of c-Jun/c-Fos subunits contained in DNA-binding complex.

The proper consensus site double strand oligonucleotides (5′-GGGACTTTCC-3′ for NF-κB, 5′-TGAGTCA-3′ for AP-1 and 5′-TTCCCGGAA-3′ for STAT3) were immobilized on ELISA microplates as bait. The nuclear fractions were incubated with the oligonucleotides for one hour, the unbound proteins were washed–out, and the DNA-bound subunits were detected with the specific primary antibody and secondary antibody conjugated with horseradish peroxidase. The results were expressed as absorbance (OD450 nm per mg of protein).

### IKKα/β activity assay

IKKα/β activity was assessed in cytosolic fractions. IKKα/β contained in cytosolic lysates was immunoprecipitated with anti-IKKα/β rabbit polyclonal antibody (Santa Cruz Biotechnology, USA), and the immunocomplex thus obtained was incubated for 30 min at 30 °C with the substrate peptides for IKK (Biotin-LDDRHDSGLDSMK), immobilized on a streptavidin-coated microplates (Reacti-BindTM Streptavidin Plate, Pierce, Rockford, IL, USA). The kinase reaction mixture contained 50 mM HEPES, pH 7.5, 20 mM MgCl_2_, 0.1 mM Na_3_VO_4_, 200 μM ATP, 10 mM β-glycerolphosphate, and 2 mM DTT. After 30 min incubation at 30 °C, the phosphorylated peptides were detected according to the standard ELISA procedure with the use of anti-p-Ser polyclonal antibody (AbD Serotec, Oxford, UK) as the primary antibody, and anti-rabbit HRP-conjugated antibody as the secondary antibody. The enzyme activity was calculated on the basis of a standard curve prepared with the phosphorylated substrate peptide (Biotin-LDDRHDpSGLDSMK) and expressed as pmol of p-IκBα per min per mg of protein.

### Statistical analysis

The statistical analysis was performed by one-way ANOVA. The statistical significance between the experimental groups and their respective controls was assessed by Tukey’s post hoc test, with *P* < 0.05 being considered significant.

## Results

### NF-κB activation in rat liver

**Fig. 1:**
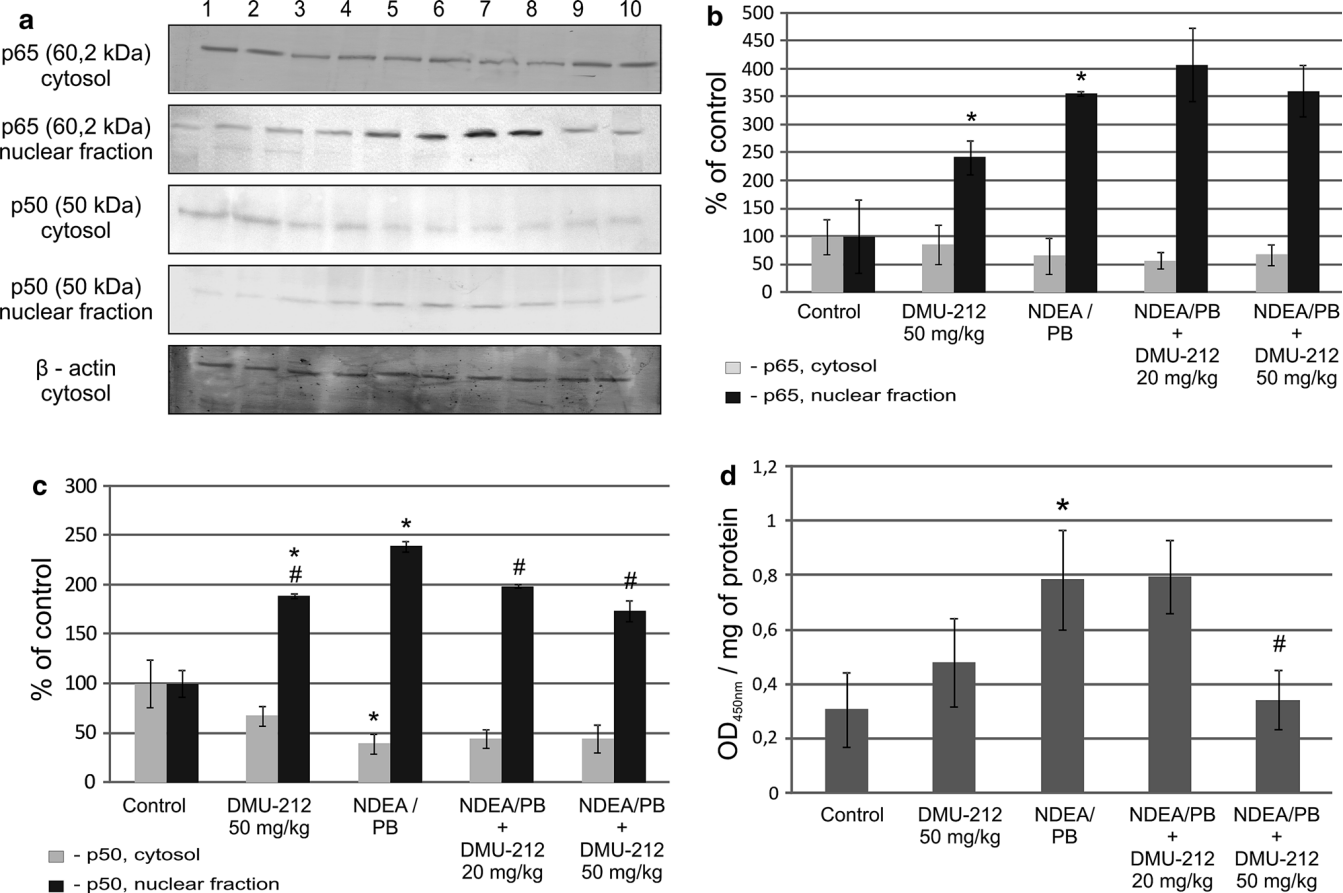
The effect of DMU-212 on NF-κB activation in rat liver translocation of p50 and p65 from cytosol to nucleus was assessed using Western blot analysis (**a**). Densitometric analysis was performed for quantitative evaluation (**b**, **c**). The blots shown are representative from two independent assays *Lane 1*, *2* control group; *3*, *4* DMU-212 50 mg/kg b.w.; *5*, *6* NDEA (200 mg/kg); *7*, *8* NDEA + DMU-212 (20 mg/kg); *9*, *10* NDEA + DMU-212 (50 mg/kg) activated NF-κB was assessed in terms of the amount of NF-κB p65 subunit contained in DNA-binding complex extracted from the nuclei isolated from liver and expressed as absorbance (OD_450nm_ per mg protein) **(d).** *Bars* represent mean ± SEM from 4 (**b**, **c**) or 5 (**d**) animals, determined by densitometric analysis (**b**, **c**) or ELISA assay (**d**). * Significantly different from control group (*P* < 0.05); ^#^ significantly different from NDEA/PB-treated group *(P* < 0.05)

Figure 1 presents the translocation of p50 and p65 subunits of NF-κB (a, b) and binding of p65 to the consensus sequence oligonucleotide (c) in various experimental rat groups in comparison with untreated control. Treatment with DMU-212 alone increased translocation of both subunits and the content of p65 in DNA-binding complex extracted from the hepatocytes nuclei (by ~150 and 50 %, respectively). This effect was lower than that observed as a result of NDEA/PB treatment protocol.

**Fig. 2:**
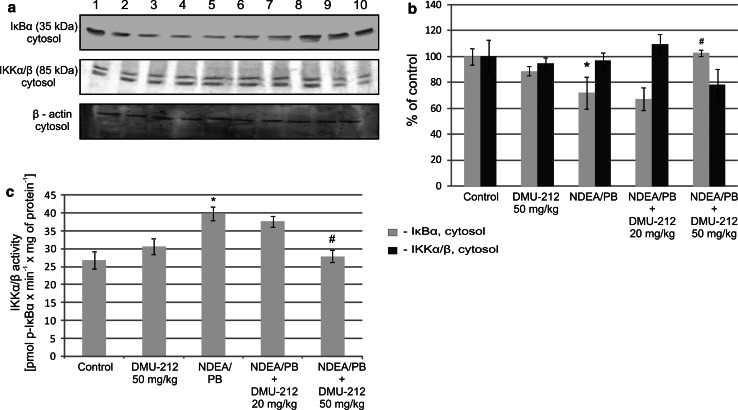
The effect of DMU-212 on IκBα retention and IKKα/β level and activity in rat liver IκBα retention and IKKα/β protein level in cytosol were assessed using Western blot analysis (**a**). The blots shown are representative from two independent assays. *Lane 1*, *2* control group; *3*, *4* DMU-212 (50 mg/kg); *5*, *6* NDEA (200 mg/kg); *7*, *8* NDEA + DMU-212 (20 mg/kg); *9*, *10* NDEA + DMU-212 (50 mg/kg). Densitometric analysis was performed for quantitative evaluation (**b**). IKKα/β activity was assayed as described in the text and is expressed as pmoles of p-IκB per minute per mg of protein (**c**). *Bars* represent mean ± SEM from 4 (**b**) or 6 (**c**) animals, * Significantly different from control group (*P* < 0.05); ^#^ Significantly different from NDEA/PB-treated group (*P* < 0.05)

Combining this protocol with DMU-12 treatment reduced the translocation of p50 subunit, while p65 content in nuclei was also slightly reduced, but the difference was not statistically significant. Treatment with a higher dose (50 mg/kg) led to a significant decrease in p65 DNA binding in comparison with NDEA/PB treated group of rats (d). Figure 2 presents the data illustrating the effect of DMU-212 on the retention of IκBα and IKKα/β protein level in cytosol (a, b) and its activity (c). The IKKα/β is responsible for IκBα phosphorylation and subsequent polyubiquitination. DMU-212 alone slightly decreased the cytosolic level of IκBα and increased the activity of IKKα/β to similar extent. These changes, however, were not statistically significant. A similar trend, but more pronounced (by ~30–50 %, respectively) was observed in the group of animals treated with NDEA/PB. The combined treatment with DMU-212 in the dose of 50 mg/kg resulted in an increased level of IκBα protein and reduced activity of IKKα/β in comparison with those in the NDEA/PB-treated group.

### AP-1 activation in rat liver

**Fig. 3:**
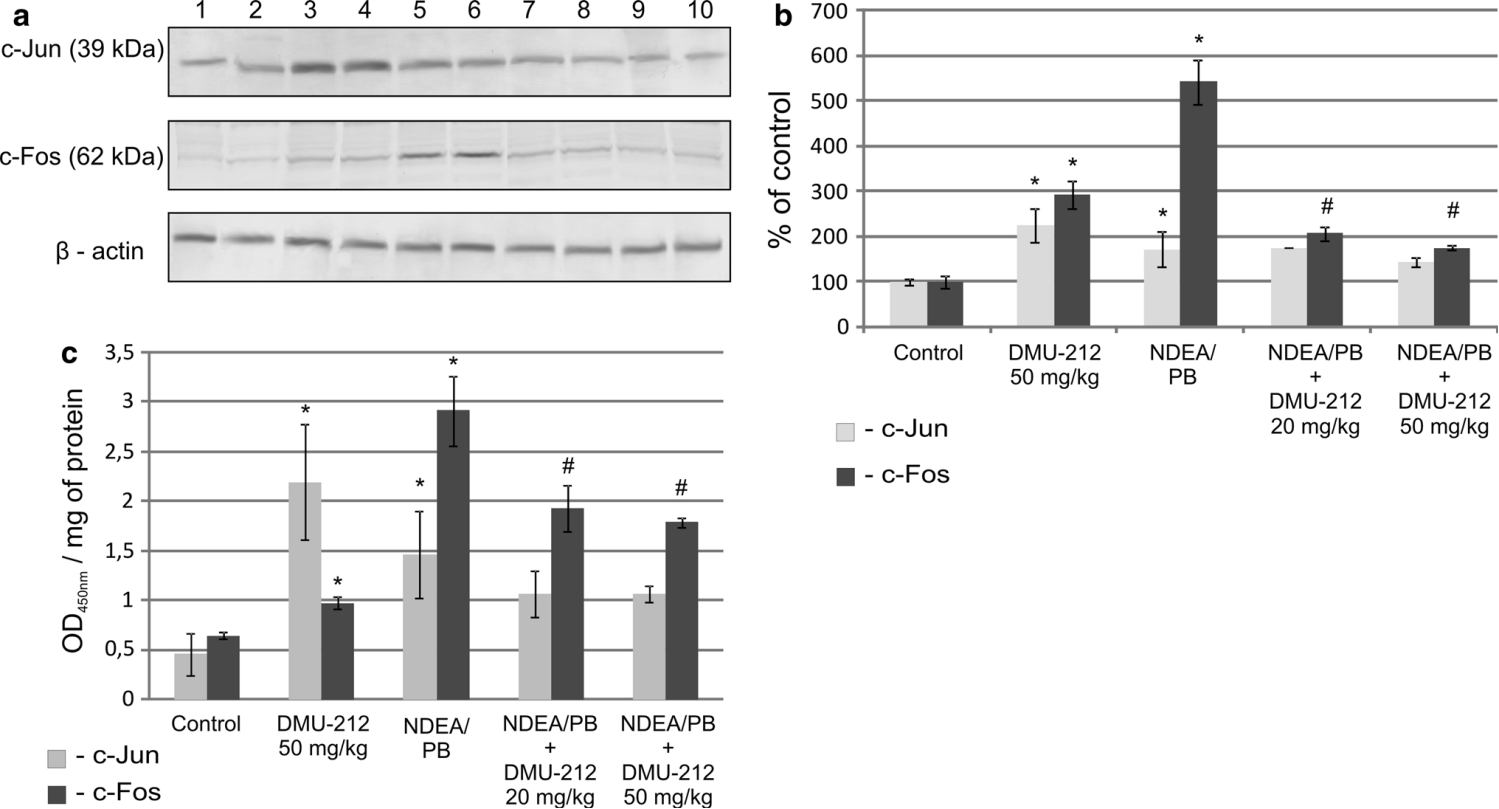
The effect of DMU-212 on AP-1 activation in rat liver protein extracts from rat liver were assayed for c-Jun or c-Fos using Western blot analysis (**a**). The Western blots shown are representative from two independent assays. *Lane 1*, *2* control group; *3*, *4* DMU-212 (50 mg/kg); *5*, *6* NDEA (200 mg/kg); *7*, *8* NDEA + DMU-212 (20 mg/kg); *9*, *10* NDEA + DMU-212 (50 mg/kg). Densitometric analysis was performed for quantitative evaluation (**b**). Activated AP-1 was assessed in terms of the amount of c-Jun or c-Fos subunits contained in DNA-binding complex extracted from the nuclei isolated from liver, and expressed as absorbance (OD_450nm_ per mg protein) (**c**). *Bars* represent mean ± SEM from 4 (**a**, **b**) or 6 (**c**) animals, determined by means of densitometric analysis (**b**) or ELISA assay (**c**). * Significantly different from control group (*P* < 0.05); ^#^ significantly different from NDEA/PB-treated group (*P* < 0.05)

AP-1 activation was evaluated in terms of the c-Jun and c-Fos subunits protein levels (Fig. 3a, b), and their amounts in the DNA binding complexes extracted from the hepatocytes nuclei (Fig. 3c). The treatment with DMU-212 alone increased both c-Jun and c-Fos levels in comparison with those in the control group of animals. Moreover, the treatment with DMU-212 increased c-Jun binding to TRE consensus site. The level of binding was significantly higher not only in comparison with the control group of animals but also with that treated with NDEA/PB. The combined treatment resulted in a decrease in both AP-1 subunits protein level and their amount in the DNA-binding complexes extracted from the hepatocytes nuclei, however, a statistically significant difference in comparison with NDEA/PB group of rats was found only for c-Fos.

### The effect of DMU-212 on COX-2 and iNOS protein levels

**Fig. 4:**
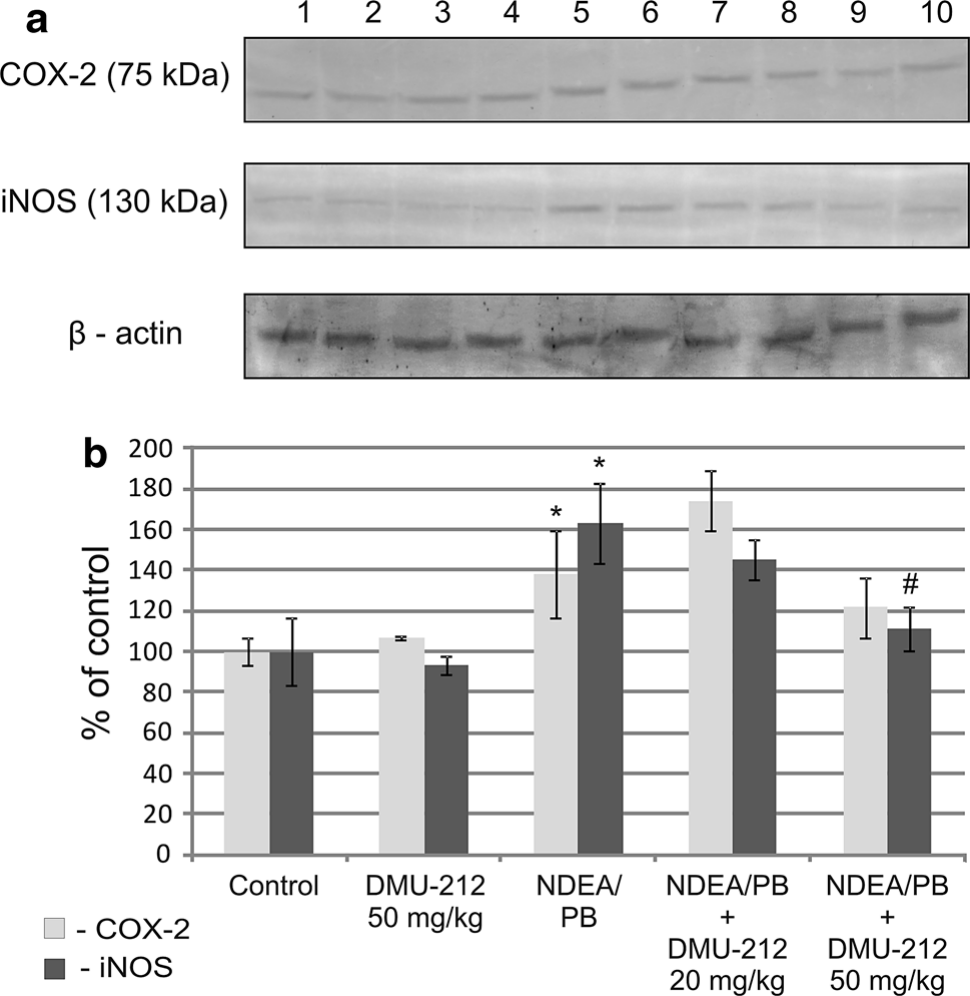
The effect of DMU-212 on COX-2 and iNOS protein level in rat liver. The levels of COX-2 and iNOS proteins in total protein lysates of rat liver were determined using Western blotting. The blots shown are representative from two independent experiments (**a**). *Lane 1*, *2* control group; *3*, *4* DMU-212 (50 mg/kg); *5*, *6* NDEA (200 mg/kg); *7*, *8* NDEA + DMU-212 (20 mg/kg); *9*, *10* NDEA + DMU-212 (50 mg/kg). Densitometric analysis was performed for quantitative evaluation (**b**). *Bars* represent mean ± SEM from 4 animals, determined by densitometric analysis. * Significantly different from control group, *P* < 0.05; ^#^ significantly different from NDEA/PB-treated group, *P* < 0.05

*COX*-*2* and *iNOS* genes are under transcriptional control of NF-κB and/or AP-1, and thus, we determined the protein level of these enzymes in the liver of rats from various treatment groups. The application of NDEA/PB treatment protocol resulted in an increase in COX-2 and iNOS protein level in liver cells lysates by ~40 and 80 %, respectively (Fig. 4). The treatment with DMU-212 alone had no effect on these enzymes protein level, while the combined treatment with the dose of 50 mg/kg decreased iNOS level as compared to that in NDEA/PB-treated rats.

### STAT3 activation in rat liver

**Fig. 5:**
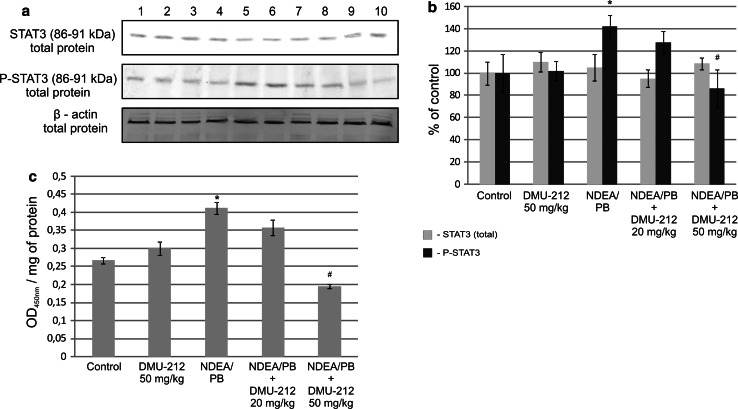
The effect of DMU-212 on STAT3 activation in rat liver. The levels of total and phosphorylated STAT3 protein in cell lysates of rat liver were determined using Western blotting. The blots shown are representative from two independent experiments (**a**). Densitometric analysis was performed for quantitative evaluation (**b**). STAT3 activation was measured in terms of the amount of STAT3 protein contained in DNA-binding complexes extracted from the nuclei of rat liver cells and expressed as absorbance (OD_450nm_ per mg protein) (**c**). *Bars* represent mean ± SEM from 4 (**b**) or 6 (**c**) animals. * Significantly different from control group, *P* < 0.05; ^#^ significantly different from NDEA/PB treated group, *P* < 0.05

Figure 5 presents the data showing the content of total and phosphorylated transcription factor STAT3 in cell lysates (a, b) and its binding to the consensus sequence oligonucleotide in various experimental rat groups (c). The NDEA/PB treatment regimen enhanced the STAT3 activation by increasing the level of its total phosphorylated form (Fig. 5b) and protein content in DNA binding complexes extracted from the nuclei of rat hepatocytes (Fig. 5c). The combined treatment with DMU-212 in both doses reduced the STAT3 activation, but the difference between both treatment groups was statistically significant only for the higher dose (50 mg/kg).

## Discussion

Resveratrol is a simple molecule that has taken the spotlight since the first scientific paper described a possible cancer preventive effects on cancer in mice. Several pre-clinical and some human intervention studies performed later have indicated that resveratrol exerts cancer chemopreventive and therapeutic responses.

However, an important issue with the future application of resveratrol for disease management is its low bioavailability due to its rapid metabolism in mammals [18], hence a strategy targeted to discover and define novel analogs of resveratrol has been assumed. These analogs should have the same structural backbone of resveratrol, with chemical modifications resulting in superior efficacy [19]. One of such analogs is DMU-212 which showed higher antiproliferative and proapoptotic activity as well as improved bioavailability in mouse liver and plasma compared to those of resveratrol [20].

The studies of Bishayee’s group have shown that resveratrol is able to inhibit NDEA-initiated and PB-promoted hepatic tumorigenesis in rats [21]. Moreover, the anti-inflammatory activity related to inhibition of iNOS induction was suggested as a possible mechanism of its anti-promotional effects [11].

The present study was thus designed to address the question whether DMU-212 affects NF-κB, AP-1, and STAT3-activated inflammatory pathways, and associated inflammatory proteins, particularly iNOS and COX-2, using a similar experimental model. We found that although DMU-212 increased the constitutive activation of NF-κB when administered in a dose of 50 mg/kg, it inhibited the NF-κB activation induced by NDEA/PB treatment. The above observations together with the increased retention of IκBα and reduced activity of IKKα/β, one of the major kinases involved in NF-κB activation pathway [22], suggest that DMU-212 affects NDEA/PB-induced NF-κB activation through inhibition of IKKs activity.

More importantly, DMU-212 applied at the same dose reduced the expression of NDEA/PB-induced iNOS, which is responsible for the production of NO, one of the major contributors to chronic inflammatory reactions. While the role of inflammation in general is considered as an important factor in HCC development, the evidence indicating that iNOS may play particularly important role in this process is also emerging. In this regard, iNOS overexpression was noted in rodent as well as in human HCC [23, 24]. Moreover, inhibition of iNOS by a selective inhibitor, aminoguanidine, led to suppression of HCC growth [24]. Thus, the results of our study indicate that similarly as for resveratrol, iNOS might be one of DMU-212 targets in prevention of HCC. The observed decrease in the NF-kB activation indicates that the reduced iNOS expression might be a result of its ability to modulate NF-kB signaling.

The above mechanism is further supported by our observation that DMU-212 did not affect the NDEA/PB-induced AP-1 activation, which might also be responsible for iNOS expression. Furthermore, iNOS overexpression during hepatocarcinogenesis was shown to be a consequence of NF-κB activation [24]. In contrast to iNOS, DMU-212 treatment had no effect on COX-2 protein level. This finding does not exclude, however, the possibility of direct DMU-212 interaction with COX-2 protein to affect the enzyme activity in this way. Such a mechanism of COX-2 modulation was suggested for resveratrol [25], although some authors, including us, have observed reduced COX-2 protein levels as a result of treatment with resveratrol [26, 27]. AP-1, in contrast to NF-κB, is minimally activated under physiological conditions, but various stimuli can dramatically increase the extent of its activation [28]. Homodimers of c-Jun/c-Jun and c-Jun/c-Fos heterodimers of AP-1 preferentially bind to the AP-1 consensus sequence TRE (TGAC/GTCA). The activity of AP-1 is regulated at the level of transcription of *c*-*jun* and *c*-*fos* genes by protein–protein interaction and also through post-translational modifications of c-Jun and c-Fos proteins [29, 30].

In our study, DMU-212 significantly increased the content of AP-1 subunits, c-Fos and c-Jun protein in rat liver extracts. Moreover, this stilbenoid enhanced the content of c-Jun in DNA-binding complex extracted from the nuclei of rat liver cells, indicating its increased binding to TRE sequence. This is an important observation since c-Jun prolonged accumulation may lead to cell cycle arrest or induction of apoptosis [31] and explain anti-proliferative and pro-apoptotic activity of DMU-212 found in earlier studies [5, 6].

On the other hand, this analog of resveratrol reduced c-Fos protein level, as well its binding to TRE consensus, increased by NDEA/PB treatment regimen. It was shown recently that derepression of c-Fos caused by microRNA-139 down-regulation contributed to the metastasis of human HCC [32]. Thus, it may be speculated that suppression of c-Fos by DMU-212 may act in the same way.

Our study showed also that DMU-212 reduced the NDEA/PB-induced activation of STAT3. Activation of STAT proteins usually occurs through phosphorylation on a specific tyrosine residue (Tyr-705 in STAT3) at the COOH terminus, but serine phosphorylation of STATs has also been demonstrated. A Pro-X-Ser-Pro sequence that is a recognition site of ERKs (extracellular signal-regulated kinase) has been found at the COOH terminus of STAT3. Moreover, it has been shown that phosphorylation on serine in DNA-binding domain of STAT3 is catalyzed by c-Jun N-terminal protein kinase (JNK) resulting in inhibition of its DNA binding and transcriptional activities [33]. Hence, it is possible that DMU-212, similarly as many other compounds, may activate JNK and thus phosphorylate STAT3 and act as a negative regulator of its activity. Such a mechanism could also explain an increased binding of constitutive c-Jun to TRE consensus as a result of treatment with DMU-212. Activated JNK phosphorylates Ser 63 and Ser 73 of c-Jun and forms a complex with the N-terminus of c-Jun, thereby protecting c-Jun from ubiquitination and subsequent degradation [34, 35].

Collectively, the results of our present study indicate that the resveratrol analog, DMU-212, may modulate the signaling pathways involved in HCC development induced by NDEA/PB initiation-promotion regimen. Although some of these activities have been demonstrated also for resveratrol, the higher bioavailability makes DMU-212 a better candidate for HCC chemopreventive agent.

## Electronic supplementary material

Below is the link to the electronic supplementary material.

Supplementary material 1 (DOC 126 kb)
