# Supplementary material for: 3,4,5,4′-trans-tetramethoxystilbene (DMU-212) modulates the activation of NF-κB, AP-1, and STAT3 transcription factors in rat liver carcinogenesis induced by initiation-promotion regimen
Source: Mol Cell Biochem. 2014 Feb 13;391(1):27–35. doi: 10.1007/s11010-014-1983-9 (PMC4006127; doi:10.1007/s11010-014-1983-9)
Supplement: Supplementary file 1 — Supplementary material 1 (DOC 126 kb) [file 11010_2014_1983_MOESM1_ESM.doc]

**ORIGINAL QUANTIFICATION NUMBERS FOR WESTERN BLOTS**

**Table 1. (fig. 1 b and c)**

| **Blot** | **Sample** | **Relative quantity** | | | | **% of control** | **SD %** |
| --- | --- | --- | --- | --- | --- | --- | --- |
|  |  | **Blot 1*** | **Blot 2*** | **Average** | **SD** |  |  |
| p65 cytosol | Control | 33,34 | 35,17 | 34,253 | 1,30 | 100,00 | 1,30 |
|  | DMU 50mg/kg | 31,98 | 28,27 | 30,123 | 2,62 | 87,94 | 2,98 |
|  | NDEA/PB | 24,88 | 20,95 | 22,915 | 2,77 | 66,90 | 4,14 |
|  | NDEA/PB+DMU 20mg/kg | 18,12 | 22,09 | 20,106 | 2,80 | 58,70 | 4,78 |
|  | NDEA/PB+DMU 50mg/kg | 26,05 | 21,45 | 23,752 | 3,25 | 69,34 | 4,69 |
| p65 nuclei | Control | 50,37 | 18,57 | 34,47 | 22,50 | 100,00 | 65,25 |
|  | DMU 50mg/kg | 100,89 | 65,40 | 83,15 | 25,10 | 241,21 | 30,19 |
|  | NDEA/PB | 126,54 | 118,66 | 122,60 | 5,57 | 355,66 | 4,54 |
|  | NDEA/PB+DMU 20mg/kg | 205,70 | 75,35 | 140,52 | 92,18 | 407,66 | 65,59 |
|  | NDEA/PB+DMU 50mg/kg | 82,70 | 165,82 | 124,26 | 58,78 | 360,48 | 47,30 |
| p50 cytosol | Control | 24,44 | 34,64 | 29,54 | 7,21 | 100,00 | 24,42 |
|  | DMU 50mg/kg | 18,42 | 21,34 | 19,88 | 2,06 | 67,30 | 10,39 |
|  | NDEA/PB | 10,76 | 12,34 | 11,55 | 1,12 | 39,10 | 9,67 |
|  | NDEA/PB+DMU 20mg/kg | 12,24 | 13,96 | 13,10 | 1,22 | 44,35 | 9,28 |
|  | NDEA/PB+DMU 50mg/kg | 11,78 | 14,32 | 13,05 | 1,80 | 44,18 | 13,76 |
| p50 nuclei | Control | 23,32 | 19,4 | 21,36 | 2,77 | 100,00 | 12,98 |
|  | DMU 50mg/kg | 39,78 | 41,04 | 40,41 | 0,89 | 189,19 | 2,20 |
|  | NDEA/PB | 49,10 | 52,94 | 51,02 | 2,72 | 238,86 | 5,32 |
|  | NDEA/PB+DMU 20mg/kg | 43,04 | 41,86 | 42,45 | 0,83 | 198,74 | 1,97 |
|  | NDEA/PB+DMU 50mg/kg | 39,98 | 34,18 | 37,08 | 4,10 | 173,60 | 11,06 |

* - average value of two spots

Table 2. (fig. 2 b)

| **Blot** | **Sample** | **Relative quantity** | | | | **% of control** | **SD %** |
| --- | --- | --- | --- | --- | --- | --- | --- |
|  |  | **Blot 1*** | **Blot 2*** | **Average** | **SD** |  |  |
| IκBα cytosol | Control | 35,25 | 32,27 | 33,76 | 2,11 | 100,00 | 6,24 |
|  | DMU 50mg/kg | 30,73 | 29,31 | 30,02 | 1,00 | 88,92 | 3,34 |
|  | NDEA/PB | 26,39 | 22,21 | 24,30 | 2,96 | 71,98 | 12,17 |
|  | NDEA/PB+DMU 20mg/kg | 24,20 | 21,26 | 22,73 | 2,08 | 67,33 | 9,14 |
|  | NDEA/PB+DMU 50mg/kg | 34,02 | 35,30 | 34,66 | 0,90 | 102,66 | 2,61 |
| IKKα/β cytosol | Control | 13,62 | 18,86 | 16,24 | 3,70 | 100,00 | 12,81 |
|  | DMU 50mg/kg | 14,98 | 15,85 | 15,42 | 0,62 | 94,90 | 4,01 |
|  | NDEA/PB | 15,00 | 16,40 | 15,70 | 0,99 | 96,65 | 6,31 |
|  | NDEA/PB+DMU 20mg/kg | 18,72 | 16,78 | 17,75 | 1,37 | 109,28 | 7,72 |
|  | NDEA/PB+DMU 50mg/kg | 14,31 | 12,13 | 13,22 | 1,55 | 78,38 | 11,70 |

* - average value of two spots

**Table 3. (fig. 3 b)**

| **Blot** | **Sample** | **Relative quantity** | | | | **% of control** | **SD %** |
| --- | --- | --- | --- | --- | --- | --- | --- |
|  |  | **Blot 1*** | **Blot 2*** | **Average** | **SD** |  |  |
| c-Jun total protein | Control | 29,69 | 26,99 | 28,34 | 1,91 | 100,00 | 6,74 |
|  | DMU 50mg/kg | 80,42 | 47,11 | 63,76 | 23,55 | 225,00 | 36,94 |
|  | NDEA/PB | 61,78 | 35,53 | 48,66 | 18,56 | 171,69 | 38,15 |
|  | NDEA/PB+DMU 20mg/kg | 49,42 | 49,23 | 49,33 | 0,13 | 174,05 | 0,27 |
|  | NDEA/PB+DMU 50mg/kg | 37,71 | 43,36 | 40,54 | 4,00 | 143,04 | 9,86 |
| c-Fos total protein | Control | 12,61 | 10,51 | 11,56 | 1,48 | 100,00 | 12,85 |
|  | DMU 50mg/kg | 40,81 | 26,80 | 33,80 | 9,91 | 292,42 | 29,31 |
|  | NDEA/PB | 41,29 | 84,06 | 62,67 | 30,24 | 542,16 | 48,25 |
|  | NDEA/PB+DMU 20mg/kg | 21,24 | 26,56 | 23,90 | 3,76 | 206,74 | 15,73 |
|  | NDEA/PB+DMU 50mg/kg | 20,95 | 19,38 | 20,16 | 1,11 | 174,44 | 5,51 |

* - average value of two spots

Table 4. (Fig. 4 b)

| **Blot** | **Sample** | **Relative quantity** | | | | **% of control** | **SD %** |
| --- | --- | --- | --- | --- | --- | --- | --- |
|  |  | **Blot 1*** | **Blot 2*** | **Average** | **SD** |  |  |
| COX-2 total protein | Control | 22,87 | 20,79 | 21,83 | 1,47 | 100,00 | 6,74 |
|  | DMU 50mg/kg | 23,52 | 23,25 | 23,38 | 0,19 | 107,12 | 0,82 |
|  | NDEA/PB | 47,51 | 30,28 | 38,90 | 12,18 | 178,18 | 31,31 |
|  | NDEA/PB+DMU 20mg/kg | 34,05 | 41,94 | 37,99 | 5,58 | 174,05 | 14,68 |
|  | NDEA/PB+DMU 50mg/kg | 25,02 | 30,66 | 27,84 | 3,99 | 127,54 | 14,33 |
| iNOS  total protein | Control | 11,27 | 14,19 | 12,73 | 2,06 | 100,00 | 16,22 |
|  | DMU 50mg/kg | 11,49 | 12,27 | 11,88 | 0,55 | 93,31 | 4,62 |
|  | NDEA/PB | 26,55 | 14,90 | 20,72 | 8,24 | 162,79 | 39,76 |
|  | NDEA/PB+DMU 20mg/kg | 16,55 | 18,98 | 17,76 | 1,72 | 139,55 | 9,67 |
|  | NDEA/PB+DMU 50mg/kg | 15,22 | 13,05 | 14,13 | 1,53 | 111,04 | 10,86 |

* - average value of two spots

**Table 5. (Fig. 5** b)

| **Blot** | **Sample** | **Relative quantity** | | | | **% of control** | **SD %** |
| --- | --- | --- | --- | --- | --- | --- | --- |
|  |  | **Blot 1*** | **Blot 2*** | **Average** | **SD** |  |  |
| STAT3 total protein | Control | 14,06 | 12,15 | 13,10 | 1,35 | 100,00 | 10,31 |
|  | DMU 50mg/kg | 13,50 | 15,31 | 14,40 | 1,28 | 109,91 | 8,89 |
|  | NDEA/PB | 12,61 | 14,93 | 13,77 | 1,64 | 105,07 | 11,88 |
|  | NDEA/PB+DMU 20mg/kg | 13,20 | 11,78 | 12,49 | 1,00 | 95,30 | 8,05 |
|  | NDEA/PB+DMU 50mg/kg | 13,72 | 14,76 | 14,24 | 0,73 | 108,63 | 5,16 |
| P-STAT3  total protein | Control | 14,14 | 17,99 | 16,06 | 2,72 | 100,00 | 16,95 |
|  | DMU 50mg/kg | 15,40 | 17,36 | 16,38 | 1,39 | 101,98 | 8,46 |
|  | NDEA/PB | 21,14 | 24,48 | 22,81 | 2,36 | 142,00 | 10,36 |
|  | NDEA/PB+DMU 20mg/kg | 21,92 | 19,08 | 20,50 | 2,01 | 127,59 | 9,80 |
|  | NDEA/PB+DMU 50mg/kg | 15,50 | 12,22 | 13,86 | 2,32 | 86,28 | 16,74 |

* - average value of two spots
